# Candidate circRNAs related to skeletal muscle development in Dazu black goats

**DOI:** 10.1080/10495398.2023.2286609

**Published:** 2023-11-30

**Authors:** Chengli Liu, Pu Yang, Xiao Wang, Baiju Xiang, Guangxin E, Yongfu Huang

**Affiliations:** a College of Animal Science and Technology, Chongqing Key Laboratory of Forage & Herbivore, Chongqing Engineering Research Centre for Herbivores Resource Protection and Utilization, Southwest University, Chongqing, China; b Chongqing Academy of Animal Sciences, Chongqing, China

**Keywords:** CircRNA, goat, muscle development, RNA_seq, host gene

## Abstract

Circular RNA (CircRNA), as a classical noncoding RNA, has been proven to regulate skeletal muscle development (SMD). However, the molecular genetic basis of circRNA regulation in muscle cells remains unclear. In this study, the expression patterns of circRNAs in the longissimus dorsi muscle at embryonic day 75 and postnatal day 1 in DBGs were investigated to identify the key circRNAs that play an important role in SMD in goats. A total of 140 significantly and differentially expressed circRNAs (DEcircRNAs) were identified among the groups at different developmental stages. Among the 116 host genes (HGs) of DEcircRNAs, 76 were significantly and differentially expressed, which was confirmed by previous RNA_seq data. Furthermore, the expression pattern of 10 DEcircRNAs with RT-qPCR was verified, which showed 80% concordance rate with that of RNA_seq datasets. Moreover, the authenticity of seven randomly selected DEcircRNAs was verified by PCR Sanger sequencing. Based on the functional annotation results, among the 76 significantly and differentially expressed HGs, 74 were enriched in 845 GO terms, whereas 35 were annotated to 85 KEGG pathways. The results of this study could provide a comprehensive understanding of the genetic basis of circRNAs involved in SMD and muscle growth.

## Introduction

Skeletal muscle is the largest tissue in animals, which accounts for nearly 40% of total body weight.^1^ Several studies have confirmed that skeletal muscle development (SMD) includes prenatal and postnatal stages. Skeletal muscle formation in mammals before birth includes proliferation, differentiation, and fusion of myogenic precursor cells (myoblasts) into multinucleated myofibers and finally the fusion of myoducts into myofibroblasts.^2^^,^^3^ However, such formation primarily includes hypertrophy of muscle fibers and repair of muscle injury after birth.^4^ In general, the two stages directly determine the formation of muscle. Interestingly, the formation of skeletal muscle and the underlying molecular mechanism during muscle development are precisely regulated by noncoding RNAs.^5^

Circular RNAs (circRNAs) are novel members of the noncoding RNA family.^6^ It often consists of exons (exonic circRNAs), but they can also arise from introns (intronic circRNAs or circular intronic RNAs).^7^ Theoretically, circRNAs resist exonuclease degradation because of their special circular structure.^8^ Meanwhile, some special circRNAs were expressed at higher levels (≥10 times) than their linear transcripts. For example, circHIPK3 originates from the second exon of the *HIPK3* gene, with higher levels than *HIPK3* mRNA.^9^ They are dynamically expressed and particularly abundant in muscle tissues across many species, including goats^5^ and sheep.^10^ In particular, circRNAs are involved in transcriptional and posttranscriptional gene expression regulation, alternative splicing, protein coding, and protein decoy.^11^^,^^12^ Recent research indicates that circRNAs play a key role in the myogenesis of various organisms.^12^ At present, most studies focus on whether circRNAs regulate SMD by sponging miRNAs. For example, several circRNAs, such as circZfp609,^13^ circFGFR4,^14^ and circFUT10,^15^ have been shown to play a role in myogenesis. Coincidentally, CDR1as and circUSP13 were found to regulate SMD in goats via the ceRNA regulatory pathway.^16^^,^^17^ In addition, circRNAs are generated from precursor mRNA back splicing.^18^ Therefore, a certain correlation could be found between a circRNA and its parental gene expression. For example, circSMARCA5 and circPAIP2 participate in the growth and development of the body by positively regulating the expression of host genes (HGs).^19^^,^^20^ Interestingly, previous studies have revealed a new role for circRNAs in regulating gene expression in the human cell nucleus, in which exon–intron circRNAs enhance the expression of their parental genes in cis and highlight a regulatory strategy for transcriptional control via specific RNA–RNA interactions between U1 snRNA and exon–intron circRNAs.^7^ However, the role that circRNAs may play in the regulation of skeletal muscle fibers in mammals remains unknown.

At the same time, some studies have shown that 60 to 90 days of embryo is a critical period for skeletal muscle cell proliferation. After birth, skeletal muscle cells are only hypertrophic and no longer proliferate.^5^ As a local goat breed in China, Dazu black goat has a high reproductive rate. However, its meat production performance is poor. Studies on muscle development in Dazu Black goat are rare. Therefore, this study systematically identified differentially expressed circRNAs (DEcircRNAs) in the longissimus dorsi muscle between the embryonic stage (75 days) and postnatal day 1 in Dazu black goat (DBG) by RNA-seq to provide a comprehensive understanding of the genetic basis of circRNAs involved in SMD.

## Materials and methods

### Biological data collection and morphological analysis of longissimus dorsi muscle tissue

In this study, 12 DBGs at the embryonic stage (75 days; ET group: E75-1, E75-2, E75-3, E75-4, E75-5, and E75-6) and postnatal day 1 (DC group: D-1, D-2, D-3, D-4, D-5, and D-6) were selected from the Southwest University farm. The whole transcriptome sequencing data of these samples were downloaded from public studies of our previous work: the RNA_seq data for circRNA identification in this research were obtained from strand-specific library RNA_seq data (Login No.: PRJNA749391). Meanwhile, significantly and differentially expressed genes involved in this study were cited from our previous work.^21^ The experimental conditions of this study were approved by the Committee on the Ethics of Animal Experiments of Southwest University (IACUC-20221128-01) and the Animal Protection Law of China.

Approximately 2–3 cm longissimus dorsi muscle of each individual was collected. It was fixed with 4% paraformaldehyde and made into paraffin sections. HE staining was performed using conventional methods, haematoxylin staining for 4 min, and eosin staining for 3 min. The morphology of skeletal muscle cells was observed under a microscope (Olympus, Japan).

### Bioinformatic analysis

The clean reads were further filtered by Fastp (version 0.18.0).^22^ The parameters were as follows: reads containing adapters were removed, reads containing more than 10% unknown nucleotides (N) were removed, and low-quality reads containing more than 50% low-quality (Q-value ≤ 20) bases were removed. At the same time, clean reads were mapped to the ribosomal RNA database by Bowtie (version 2.2.8).^23^ Then, rRNA was deleted and mapped to the reference genome ARS1 (http://asia.ensembl.org/Capra_hircus/Info/Index) by Hisat2 (version 2.1.1).^24^ The unmapped reads were then collected to identify circRNAs by find_circ software.^25^ A candidate circRNA was called if it was supported by at least two unique back-spliced reads in at least one sample.

### Verification of the accuracy of RNA_seq and identification of candidate circRNAs

All primers of full-length sequences of candidate circRNAs (Table S1) and RT–qPCR (Table S2) were designed using the Primer3plus online tool to identify the accuracy of RNA_seq data (http://www.primer3plus.com/cgi-bin/dev/primer3plus.cgi, March 14, 2022), and the primers were synthesized by Wuhan Tianyi Huiyuan Biological Technology Co., Ltd. (Wuhan, China; Tables S1 and S2). Total RNA was converted to cDNA using the Transcriptor First Strand cDNA Synthesis Kit (TAKARA, Japan).

PCR (50 μL) consisted of 3.0 μL of cDNA, 2.5 μL of forward primer, 2.5 μL of reverse primer, 17.0 μL of RNase-free water, and 25.0 μL of Megafi™ Fidelity 2 × PCR MasterMix (ABM, Canada) in obtaining the full-length sequences of candidate circRNAs. The amplification procedure was as follows: 98 °C for 2 min, 98 °C for 15 s, Tm°C for 30 s, 72 °C for 40 s, and 72 °C for 5 min. PCR products were detected by agarose gel electrophoresis. Meanwhile, PCR products underwent Sanger sequencing by ABI3730 (AB, USA), and MAGA 4^26^ was used for sequence alignment and splicing.

RT-qPCR (15 μL) consisted of 1.0 μL of cDNA, 0.4 μL of forward primer, 0.4 μL of reverse primer, 7.5 μL of TB Green Premix Ex TaqII (TAKARA, Japan), and 5.7 μL of ddH_2_O in identifying the accuracy of RNA_seq data. The amplification procedure was as follows: 94 °C for 10 min, 94 °C for 30 s, 60 °C for 30 s, and 72 °C for 40 s. The transcripts were quantified using standard curves with 10-fold serial dilutions of cDNA. Melting curves were constructed to verify whether only a single PCR product was amplified. The samples within runs were assayed in triplicate, with standard deviations of the threshold cycle (Ct) values not exceeding 0.5; each qPCR run was repeated at least three times. Negative (without template) reactions were performed within each assay. Statistical significance of qPCR was determined by ANOVA.

### Functional analysis

DEcircRNAs (FDR < 0.001 and |log(FC)|>2) between DC and ET were identified by edgeR (version 3.12.1; http://www.r-project.org/).^27^ Furthermore, Gene Ontology (GO) functional enrichment and Kyoto Encyclopedia of Genes (KEGG) analysis were performed on DE_HGs by KOBAS (http://kobas.cbi.pku.edu.cn/genelist/, August 19, 2022).

## Results and analysis

### Histomorphological observation of the longissimus dorsi muscle of the DBG at ET and DC

**Figure 1.**
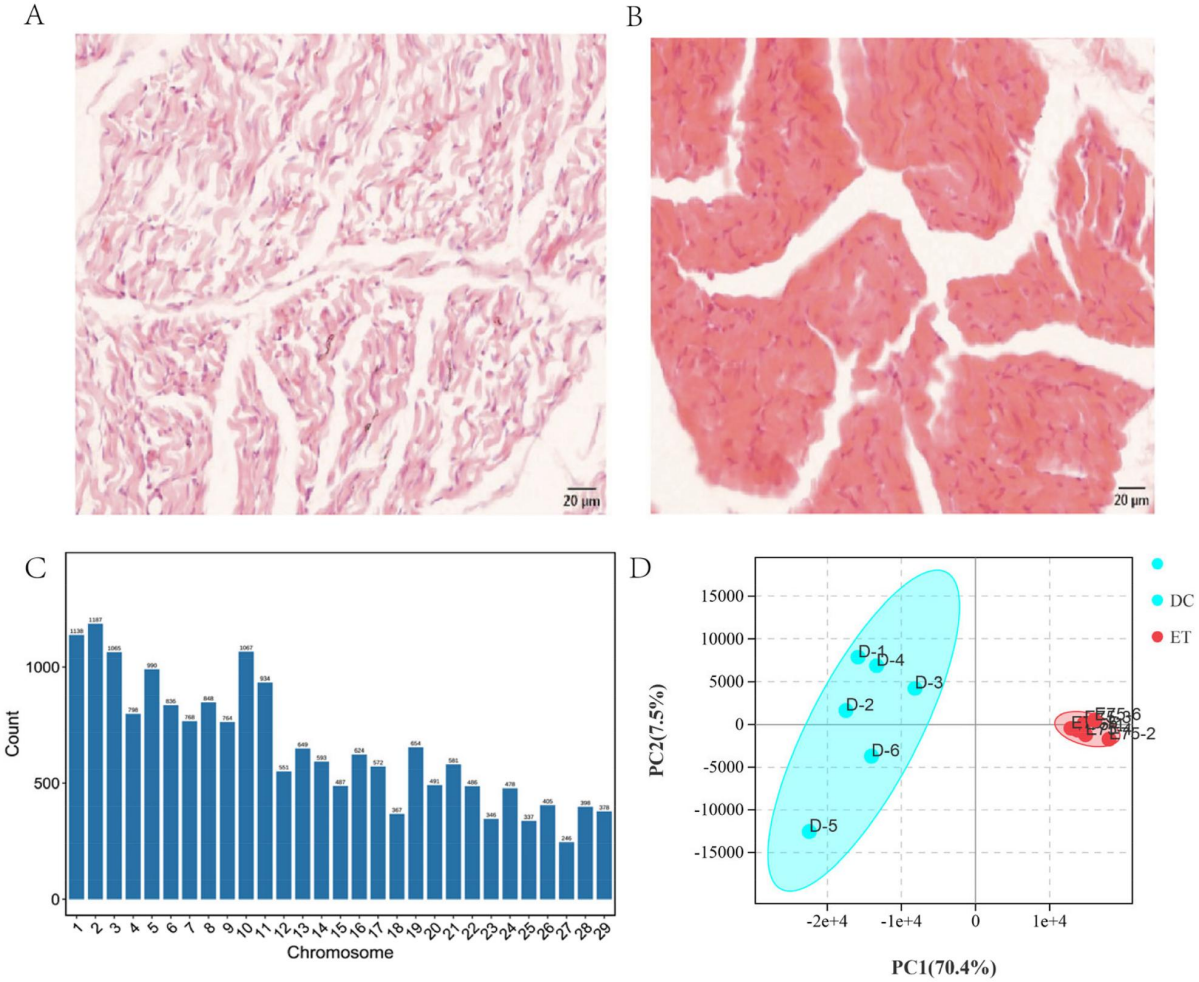
Histomorphology of longissimus dorsi muscle and circRNAs distribution analysis. (A), HE staining of longissimus dorsi tissue of DBG embryo at day 75; (B), HE staining of longissimus dorsi tissue of DBG 1 day after birth; (C), distribution of all identified circRNAs on chromosomes; (D), Principal component analysis (PCA) was performed on all identified circRNAs in the longissimus dorsi muscle of 12 DBGs.

Based on the histomorphological results, the tissue sections of the ET stage contained more primary muscle fibres than those of the DC stage (Figure 1(A)). Conversely, no primary muscle fibres were found in the DC stage, and all muscle cells were secondary fibres (Figure 1(B)). These results indicate that embryonic day 75 and postnatal day 75 are two different stages of skeletal muscle development in goats (Table 1)

**Table 1.** Comparison of abbreviations and full names.

Abbreviations	Full Names
DBG	Dazu black goat
ET	experimental team
DC	control group
DE	Significantly differently expressed
mRNA	messenger RNA
circRNA	circular RNA
ncRNA	noncoding RNA
DEcircRNAs	Significantly differently expressed circRNAs
RPM	back-spliced reads per million mapped reads
DE_HG	Significantly differently expressed host genes
log2FC	log2 (fold change)
KEGG	kyoto encyclopedia of genes and genomes
GO	gene ontology
SMD	skeletal muscle development
FDR	false discovery rate
RT-qPCR	reverse transcription quantitative PCR
*LMO7*	LIM domain 7
*IGF2BP3*	insulin like growth factor 2 mRNA binding protein 3
*CDC6*	cell division cycle 6
*FTO*	FTO alpha-ketoglutarate dependent dioxygenase
*EZH2*	enhancer of zeste 2 polycomb repressive complex 2 subunit
*BCAT1*	branched chain amino acid transaminase 1
*CD36*	CD36 molecule
*CKM*	creatine kinase M-type
*HACD1*	3-hydroxyacyl-CoA dehydratase 1
*CD36*	*CD36 molecule*
*MyoD*	myogenic differentiation 1
*AMPK*	AMP-activated protein kinase
HGs	host genes
RNA-seq	RNA sequencing
C2C12	mouse myoblast

### Prediction and characterization of circRNAs at different stages of SMD in goat

With regard to RNA_seq data, an average of 95.4 million reads were obtained from 12 cDNA libraries (Tables S3 and S4). A total of 19,038 circRNAs and 5131 HGs were obtained (Table S5). The chromosome distribution of circRNAs showed that most circRNAs were produced from chromosomes 1 and 2 (Figure 1(C)). Meanwhile, circRNA-type analysis showed that Annot_exons were the most common sequence, with an average proportion of 72.7%. In this study, the length range of circRNAs was 70–99435 bp, and 34.57% of circRNA length sizes were concentrated in the range of 0–500 bp (Table S5).

### Expression analysis of circRNA

**Figure 2.**
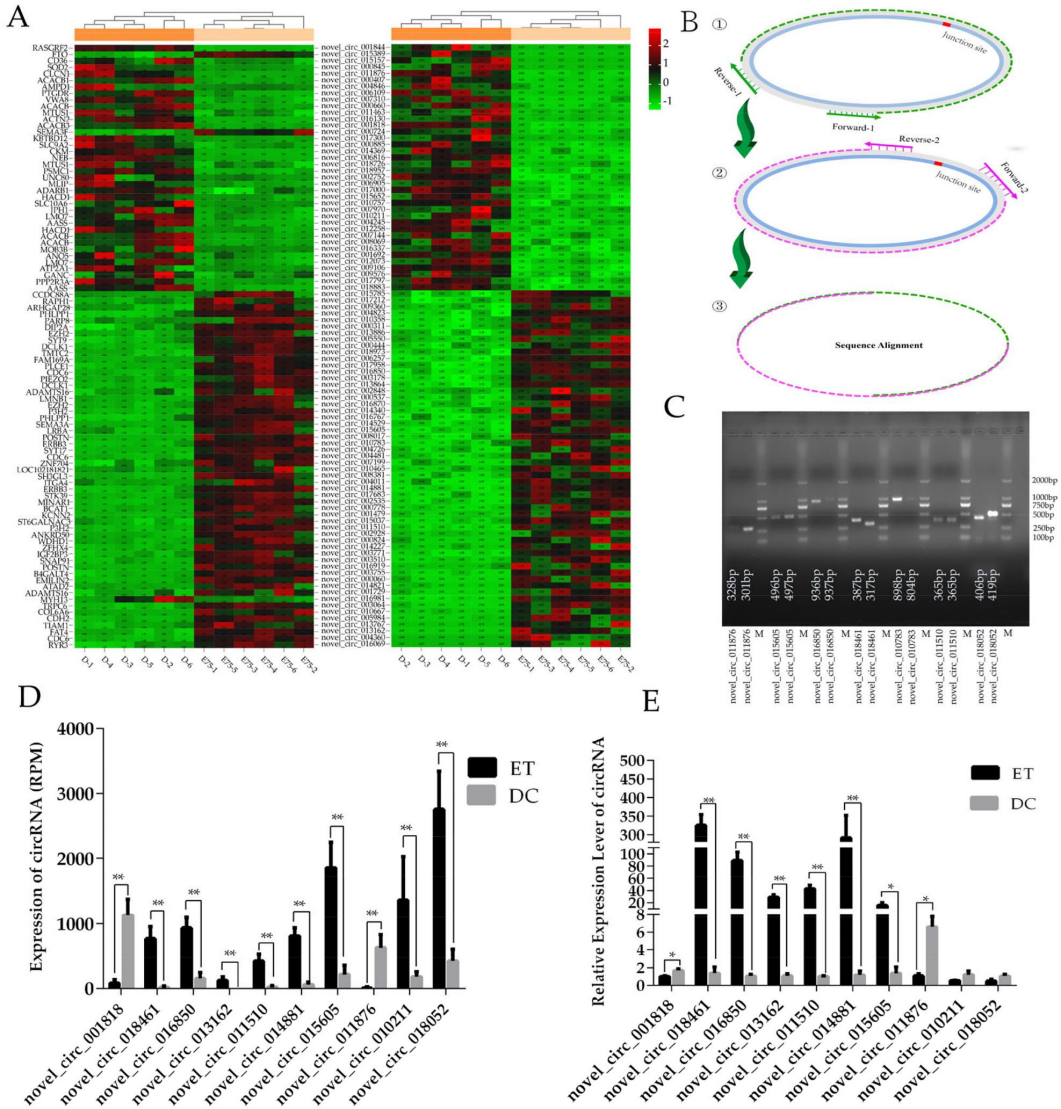
Analysis of circRNA expression in two stages of skeletal muscle development of DBG: (a), clustering heatmap of DEcircRNA and DE_HGs; (B), schematic diagram of obtaining the full length of circRNA by PCR amplification; (C), agarose gel electrophoresis of PCR amplification products; (D), expression levels of circRNA (RPM) by RNA_seq; (E), relative expression level of circRNA by RT_qPCR; “*”: *P* < 0.05; “**”: *P* < 0.01.

Whether circRNAs participate in goat SMD must be verified. Principal component analysis of all circRNAs in 12 samples showed that ET and DC samples were two independent clusters, which suggests that the differentially expressed pattern of circRNAs in the two stages of SMD (Figure 1(D)) and circRNAs may be involved in the development of goat skeletal muscle. Therefore, we further screened 140 DEcircRNAs (FDR <0.001 and |log (FC)|>2) and 116 HGs in the two stages of SMD of DBGs (Table S6). A total of 76 HGs were significantly and differentially expressed between ET and DC from the RNA_seq dataset (Figure 2(A) and Table S7).

To confirm the authenticity of circRNAs, which was predicted by RNA_seq data, the PCR-amplified full length of seven DEcircRNAs was displayed by PCR Sanger sequencing, which confirmed their existence. The length and nucleic acid composition of six DEcircRNAs were completely consistent with those of the RNA_seq results. However, the length of novel_circ_010783 was 130 nt more than that of the RNA_seq result at the junction site (Figure 2(B,C)). Furthermore, the RNA expression level of 10 DEcircRNAs based on the RT-qPCR results was consistent with the expression trends observed in the RNA_seq data, and 80% of circRNAs were significantly and differentially expressed. This finding indicates that the RNA_seq result is reliable (Figure 2(D,E)).

### Functional analysis of DE_HGs

**Figure 3.**
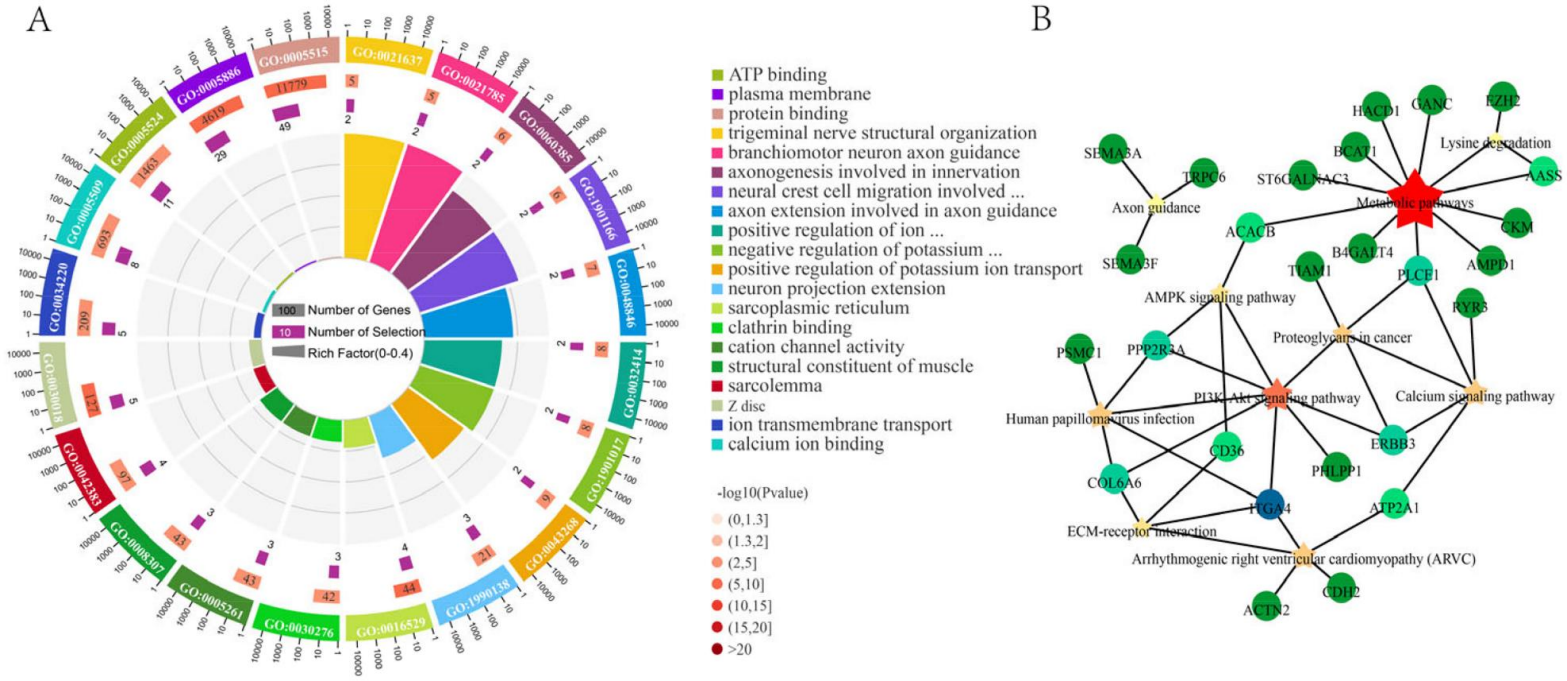
Functional analysis of DEcircRNA in two stages of skeletal muscle development of DBG: (A), top 20 pathways of GO enrichment analysis; (B), top 10 pathways of KEGG enrichment analysis.

In this study, the expression trend of 96.8% DEcircRNAs was consistent with its HGs. This finding suggests that circRNAs may play a biological function by regulating the expression of HGs. Therefore, 76 HGs were subjected to functional analysis. The GO analysis results showed that 74 DE_HGs were enriched in 845 GO terms, among which 459 GO terms were significantly enriched (*P* < 0.05), including the regulation of cell population proliferation, muscle tissue morphogenesis, and a large number of muscle-related GO terms (Figure 3(A) and Table S8). In addition, more than 50 GO terms were involved in mitosis, including cell division, DNA replication, and regulation of the cell cycle. Further analysis revealed that several genes were enriched in many GO terms related to the regulation of cell proliferation, death, and apoptosis. These genes play important roles in animal SMD.

The KEGG results revealed that 35 DE_HGs were enriched in 85 KEGG pathways, 28 of which were significantly enriched (*P* < 0.05), such as metabolic pathways, the calcium signaling pathway, and the PI3K-Akt signalling pathway (Figure 3(B) and Table S9). Among them, metabolic pathways had the most enriched DE_HGs, including 10 enzymes associated with metabolism. Meanwhile, some of these DE_HGs have been confirmed to be involved in myoblast fusion, muscular dystrophy, cell proliferation, and skeletal muscle growth.

## Discussion

The chromosome distribution of circRNAs showed that most circRNAs were produced from chromosomes 1 and 2 (Figure 1(C)), which is consistent with that of a previous study on goat skeletal muscle.^28^ However, the opposite conclusion shows that the number of circRNAs on chromosomes 1 and 3 was higher than that on other chromosomes in the skeletal muscles of Anhui White goats.^5^ Therefore, the expression of circRNAs is specific to breeds and tissues. Meanwhile, circRNA-type analysis showed that Annot_exons were the most common sequence, with an average proportion of 72.7%. This result is consistent with circRNA species found in the muscle of numerous mammals, such as pig,^29^^,^^30^ cow,^31^ and chicken.^32^ In this study, the length range of circRNAs was 70–99435 bp, and 34.57% of circRNA length sizes were concentrated in the range of 0–500 bp (Table S5). This result is similar to the circRNA length ratio of other goat breeds.^28^ However, the circRNA length ranged from 35 to 1,866 bp, and most (94.97%) circRNA lengths were less than 500 bp in Wuan goats.^33^ Therefore, the length distribution and type of circRNAs may also be breed specific.

The expression level of circRNAs is directly related to their function. In this study, novel_013326, which is derived from the LIM domain 7 gene (*LMO7*), had the highest expression level in the two stages of SMD. Notably, the circRNA (circ_001086) with the highest expression level is also derived from *LMO7* in the longissimus dorsi muscle of Liaoning cashmere goats and Ziwuling Black goats at 9 months of age.^28^ However, circITSN2 can promote the proliferation and differentiation of chicken primary myoblasts through the miR-218-5p/*LMO7* axis.^34^ Moreover, the knockdown of *LMO7* resulted in a reduction in myoblasts and myotubes in primary cultures of embryonic chick muscle cells. Interestingly, the *LMO7* gene is significantly and differentially expressed in DBGs at two stages of SMD.^21^ All the above-mentioned results indicate that *LMO7* may play a role in SMD in goats, but its mechanism needs to be further studied.

Functional analysis showed that a large number of genes were enriched in GO terms related to skeletal muscle development。These results indicate that the *IGF2BP3*, *CDC6*, and *EZH2* genes may be involved in the proliferation of skeletal muscle cells in goat embryos. In this study, the expression of these genes in ET was significantly higher than that in DC.^21^ For example, studies have found that fat mass and obesity-associated protein (*FTO*) gene expression were closely related to the differentiation of mouse myoblast (C2C12) myoblasts, and SMD is impaired in *FTO*-deficient mice.^35^ Moreover, circPPP1R13B positively regulated the expression of the miR-9-5p target gene insulin-like growth factor 2 mRNA-binding protein 3 (*IGF2BP3*) and further activated the downstream insulin-like growth factor (*IGF*)/phosphatidylinositol 3-kinase (PI3K)/AKT serine/threonine kinase (AKT) signalling pathway.^36^ The proliferation and differentiation of chicken skeletal muscle satellite cells were further regulated.^36^ Meanwhile, let-7b directly inhibited *IGF2BP3* expression by binding to its 3′UTR, which inhibited cell proliferation and induced cell cycle arrest in chicken myoblasts.^37^ Furthermore, myogenic differentiation 1 (*MyoD*) can occupy an E-box within the division cycle 6 promoter (*CDC6*) in C2C12 and murine primary myoblasts, and *MyoD* knockdown can impair the ability of C2C12 cells to express *CDC6* after leaving quiescence, which resulted in C2C12 cells not fully progressing to S-phase.^38^ Moreover, the enhancer of the zeste homolog 2 (*EZH2*) gene was found to be essential for cell survival, and the moderate inhibition of *EZH2* resulted in inhibited skeletal muscle cell proliferation and S-phase cell accumulation.^39^ These studies showed that *IGF2BP3*, *CDC6*, and *EZH2* could promote cell proliferation, and the proliferation of goat skeletal muscle cells primarily occurred in the ET phase.^3^

In addition, creatine kinase M-type (*CKM*) is an M-type isoenzyme of creatine kinase (*CK*).^40^ However, *CK* is a key enzyme in energy metabolism during skeletal muscle contraction and relaxation, and it is involved in mitochondrial oxidative phosphorylation.^41^ Similarly, 3-hydroxyacyl-CoA dehydratase 1 (*HACD1*) deficiency in humans and dogs leads to congenital myopathy with fibre size disproportion associated with generalized muscle weakness.^42^ *HACD1*-null promotes myoblast fusion during muscle development and regeneration in Labrador, mice, and myoblasts.^43^ In addition, the branched-chain amino acid transaminase 1 (*BCAT1*) gene helps muscle cell growth by activating mTOR signalling and reducing the production of reactive oxygen species.^44^ These results indicate that *HACD1*, *CKM*, and *BCAT1* may be involved in SMD by regulating the metabolism of various organic compounds.

Subsequently, insulin resistance,^45^ the AMP-activated protein kinase (AMPK) signaling pathway,^46^ and the thyroid hormone signalling pathway^47^ have been confirmed to be involved in SMD. In particular, the AMPK signalling pathway is widely recognized to regulate apoptosis, differentiation, and nutrient uptake of muscle fibres in humans,^48^ pigs,^49^ ducks,^50^ and chickens.^51^ For example, AMPK is a master regulator of energy metabolism in skeletal muscle; AMPK activation increases NAD^+^ levels in C2C12 myoblasts and skeletal muscle.^52^ Meanwhile, *AMPK* regulates downstream *SIRT1* activity by increasing NAD^+^ levels.^53^^,^^54^ Similarly, the upregulation of *AMPK* and *SIRT1* is involved in the switch of skeletal muscle fibre type from fast twitch to slow twitch.^55^ AMPK has been found to inhibit muscle growth by stimulating the expression of myostatin in C2C12 cells.^56^ Therefore, the AMPK signalling pathway may play a role in goat SMD through multiple pathways in muscle cells. In this study, the *CD36* molecule (*CD36*) was enriched in the AMPK signaling pathways. Some studies have shown that *CD36* expression is significantly upregulated during myoblast differentiation, and endogenous *CD36* knockdown significantly decreases the expression of myogenic markers and myotube formation.^57^ Furthermore, *CD36* has been shown to be a key mediator of fatty acid uptake in skeletal muscle, and *CD36* knockdown prevents diet-induced obesity, intramuscular lipid deposition, and oxidative stress, which results in impaired muscle satellite cell function and delayed muscle regeneration.^58^^,^^59^ However, the *CD36* gene can produce four circRNAs in the two stages of goat SMD, and novel_circ_015157 is highly and differentially expressed (FDR < 0.001). Therefore, SMD in goats may be precisely regulated by *CD36* and circRNAs.

Notably, novel_circ_010783 and its HG (*ERBB3*) were highly expressed in ET. Nearly no detectable dose was observed in the DC group. Several studies have found that the *ERBB3* gene is primarily involved in SMD through the ErbB signalling, MAPK signalling, and PI3K-Akt signalling pathways.^60^^,^^61^ For example, JNK/MAPK signalling has been demonstrated to inhibit skeletal muscle differentiation by negatively regulating *MyoD.*^62^ Furthermore, *VEGFB* promotes the proliferation of C2C12 myoblasts through the VEGFR1-PI3K/Akt signalling pathway.^63^ In summary, the ways of circRNA involved in skeletal muscle development are diverse, and the specific mechanisms need to be further studied.

## Conclusion

We analysed the expression pattern of circRNAs in the longissimus dorsi muscle of goats at embryonic day 75 and postnatal day 1 in DBGs. Meanwhile, circRNAs such as novel_013326 and novel_circ_010783 were found to be involved in goat skeletal muscle development through ErbB, MAPK, and PI3K-Akt signalling pathways. These results will provide a basis for exploring the molecular mechanism of circRNA and regulating SMD and lay a foundation for molecular-assisted breeding of meat goats.

## Supplementary Material

Supplemental Material

## Data Availability

All data of RNA_seq data was cited and download from NCBI database (PRJNA749391).
